# Elucidation of degrading pattern and substrate recognition of a novel bifunctional alginate lyase from *Flammeovirga* sp. NJ-04 and its use for preparation alginate oligosaccharides

**DOI:** 10.1186/s13068-019-1352-8

**Published:** 2019-01-10

**Authors:** Benwei Zhu, Fang Ni, Yun Sun, Limin Ning, Zhong Yao

**Affiliations:** 1 College of Food Science and Light Industry, Nanjing Tech University, 30 Puzhu Rd, Nanjing, 211816 People’s Republic of China; 2 College of Medicine and Life Science, Nanjing University of Chinese Medicine, Nanjing, 210023 Jiangsu China

**Keywords:** Alginate lyase, Bifunctional, Oligosaccharide, Action pattern, Substrate recognition

## Abstract

**Background:**

The alginate oligosaccharides have been widely used in agriculture, medicine, and food industries due to their versatile physiological functions such as antioxidant, anticoagulant, and antineoplastic activities. The bifunctional alginate lyases can degrade the alginate polysaccharide more efficiently into alginate oligosaccharides. Therefore, it is crucial to discover new bifunctional alginate lyase for alginate oligosaccharide production.

**Results:**

Herein, a novel bifunctional alginate lyase FsAlgB was cloned and identified from deep-sea bacterium *Flammeovirga* sp. NJ-04, which exhibited broad substrate specificity and the highest activity (1760.8 U/mg) at pH 8.0 and 40 °C. Furthermore, the *K*_m_ values of FsAlgB towards polyG (0.69 mM) and polyMG (0.92 mM) were lower than that towards sodium alginate (1.28 mM) and polyM (2.06 mM). Recombinant FsAlgB was further characterized as an endolytic alginate lyase, and it can recognize the tetrasaccharide as the minimal substrate and cleave the glycosidic bonds between the subsites of − 3 and + 1.

**Conclusion:**

This study provided extended insights into the substrate recognition and degrading pattern of alginate lyases with broad substrate specificity.

**Electronic supplementary material:**

The online version of this article (10.1186/s13068-019-1352-8) contains supplementary material, which is available to authorized users.

## Background

Alginate is the main component of the cell wall of brown algae such as *Laminaria japonica* [1]. It is a linear acidic polysaccharide consisting of β-d-mannuronate (M) and its C5 epimer α-l-guluronate (G). These units are arranged into three different kinds of blocks, namely poly β-d-mannuronate (polyM), poly α-l-guluronate (polyG) and the heteropolymer (polyMG) [2]. As the most abundant marine biomass and low-cost material, alginate has been widely used in food and medical industries due to its favorable chemical properties and versatile activities. Insufficiently, the applications of this linear polysaccharide have been greatly limited due to its high molecular weight and low bioavailability. Recently, the degradation products of alginate, namely the alginate oligosaccharides, have attracted increasing attention due to their biological activities and excellent solubility. For instance, they have been widely used as plant growth accelerators, anticoagulants, and tumor inhibitors due to their versatile physiological functions such as antioxidant, anticoagulant, and antineoplastic activities [3–5]. Moreover, they can also regulate the blood sugar and lipid content [6]. As a result, it is more promising to prepare the functional oligosaccharides by utilizing the abundant polysaccharides.

Alginate lyase and can degrade alginate into unsaturated oligosaccharides by β-elimination [7]. On the basis of their substrate specificities, alginate lyase can be classified into G block-specific lyase (polyG lyase, EC4.2.2.11) and M block-specific lyase (polyM lyase, EC4.2.2.3) [8]. While according to the mode of action, it can be sorted into endolytic and exolytic ones [9]. Endolytic enzymes can recognize and cleave glycosidic bonds inside alginate polymers with unsaturated oligosaccharides (such as di-, tri-, and tetra-saccharides) as main products [10], while exolytic ones can further degrade oligosaccharides into monomers [11]. In addition, according to protein sequence similarity, alginate lyases are organized into the PL-5, 6, 7, 14, 15, 17, and 18 families (http://www.cazy.org/fam/acc_PL.html) [12].

So far, alginate lyases have been widely used to elucidating fine structures of the alginate and prepare protoplast [13, 14]. Moreover, it also shows great potential in treating cystic fibrosis by degrading the polysaccharide biofilm of pathogen bacterium such as *Pseudomonas aeruginosa* [15, 16].

In this study, we cloned and identified a novel bifunctional alginate lyase of PL7 family FsAlgB. Like most of alginate lyases of PL 7 family, it can degrade alginate into oligosaccharides in an endolytic manner [17]. It is reported that the A1-II′ from *Sphingomonas* sp. A1, a member of PL 7 family, displays a β-sandwich jelly roll-fold, and can bind tetrasaccharide as its minimal substrate and hydrolyzed it into disaccharide and trisaccharide. The structure of A1-II′ complexed with tetrasaccharide indicated that Gln_189_ and Arg_146_ act as a neutralizer for the substrate carboxyl group, His_191_ as a general base, and Tyr_284_ as a general acid [18]. However, the substrate degradation mode of PL 7 family enzymes is still unclear. The *Flammeovirga* sp. NJ-04 is an alginate-degrading bacterium isolated from deep-sea area and a gene cluster for degrading alginate has been identified [19]. In this paper, the degradation mode of FsAlgB was discussed, and the biochemical characteristics, action mode, and product analysis were also studied.

## Methods

### Materials and strains

Sodium alginate from *Macrosystis pyrifera* (*M*/*G* ratio 77/23) was purchased from Sigma-Aldrich (viscosity ≥ 2000 Cp, St. Louis, MO, USA). PolyM and polyG (purity: about 95%; M/G ratio: 97/3 and 3/97; average degree of depolymerization (DP): 39; average molecular weight: 7200 Da) were purchased from Qingdao BZ Oligo Biotech Co., Ltd (Qingdao, China). PolyMG (M/G ratio 48/52, average DP: 50, and average molecular weight: 8000 Da) was donated by the bioengineering group of our college. Marine bacterium *Flammeovirga* sp. NJ-04 was previously isolated from South China Sea and conserved in our laboratory [20]. *Escherichia coli* DH5α was used for plasmid construction, and *E. coli* BL21 (DE3) was used for gene expression. These two strains were grown in Luria–Bertani (LB) broth or on LB broth agar plates (LB broth supplemented with 1.5% agar) containing 100 μg/mL ampicillin.

### Sequence analysis

The conserved domains of FsAlgB were predicted using the InterProScan 4 running the HMM Pfam application (http://www.ebi.ac.uk/Tools/pfa/iprscan/). The homology protein sequence was performed with NTI vector. The phylogenetic tree was constructed based on related alginate lyase protein sequences of PL 7 family using Molecular Evolutionary Genetics Analysis (MEGA) Program version 6.0. The homology modeling and docking was built by Protein Homology/analogY Recognition Engine V 2.0.

### Expression and purification of FsAlgB

As previously reported, the strain *Flammeovirga* sp. NJ-04 was identified to be close to *Flammeovirga* sp. OC4 [20]. Therefore, the primers for cloning *FsAlgB* were designed on the basis of sequence of putative alginate lyase gene sequence (WP_044204802.1) within the genome of *Flammeovirga* sp. OC4. The *FsAlgB* gene was amplified with primers designed as follows: the forward primer: 5′-GGCCATATGATGAACAGACTTTTTACTTT-3′ and the reverse primer: 5′-GGCCTCGAGTTGATGTGTTACCGACAAGT-3′ from the genomic DNA of *Flammeovirga* sp. NJ-04.

The alginate lyase gene was subcloned and ligated into pET-21a (+) expression vector. The recombinant *E. coli* BL21 (DE3) harboring the pET-21a (+)*/FsAlgB* was cultured in an LB medium (containing 100 μg ampicillin/mL) for 2–3 h with shaking at 200 rpm and 37 °C up to an OD_600_ of 0.4–0.6. The cells were induced by adding 0.1 mM IPTG and then cultured at 20 °C for 30 h. The purification of FsAlgB was carried out as previously described as follows [20]. The cell homogenate containing the recombinant protein was loaded onto Ni–NTA Sepharose column (GE Healthcare, Uppsala, Sweden) equilibrated with lysis buffer. The column was washed with wash buffer [50 mM Tris-HCl (pH 8.0), 300 mM NaCl, and 20 mM imidazole], and the recombinant enzyme was eluted with elution buffer [50 mM Tris-HCl (pH 8.0), 300 mM NaCl, and 300 mM imidazole]. The active fraction was collected and desalted using HiTrap™ desalting column (Amersham Biosciences, Buckinghamshire, UK) and then analyzed by 12% sodium dodecyl sulfate polyacrylamide gel electrophoresis (SDS-PAGE).

### Substrate specificity and enzymatic kinetics

The activity of FsAlgB was determined according to the ultraviolet absorption method described by Inoue [21]. The reaction system was constructed as follows: the purified enzyme (0.1 mL 50 mM Tris-HCl buffer of pH 8.0 containing 0.042 mg of enzyme) was mixed with 0.9 mL substrate in 50 mM Tris-HCl buffer with 1% sodium alginate (pH 8.0) and the mixture was incubated at 40 °C for 10 min. The reaction was stopped by heating in boiling water for 10 min. The enzyme activity was then determined by measuring the increased absorbance at 235 nm. One unit was defined as the amount of enzyme required to increase the absorbance at 235 nm by 0.1 per min. Each experiment consisted of three replicates. In addition, the protein concentrations were determined by a protein quantitative analysis kit (Beyotime Institute of Biotechnology, Nantong, China).

To investigate the substrate specificity, the purified FsAlgB (~ 0.19 ng) in 1 mL of 50 mM Tris-HCl buffer of pH 8.0 was reacted with 1% of sodium alginate, polyMG, polyM, and polyG, respectively. The assay of enzyme activity was defined as described previously. The kinetic parameters of the FsAlgB towards these four kinds of substrates were determined by measuring the enzyme activity with substrates at different concentrations (0.1–8.0 mg/mL). The concentrations of the substrates and velocity (*V*) were calculated as previously reported [20]. The concentrations of the product were determined by monitoring the increase in absorbance at 235 nm using the extinction coefficient of 6150 M^−1^ cm^−1^. Velocity (*V*) at the tested substrate concentration was calculated as follows: *V* (mol/s) =  (milliAU/min × min/60 s × AU/1000 milliAU × 1 cm)/(6150 M^−1^ cm^−1^) × (2 × 10^−4^ L). The *K*_m_ and *V*_max_ values were calculated by hyperbolic regression analysis as previously described [22, 23]. The turnover number (*k*_cat_) of the enzyme was calculated by the ratio of *V*_max_ versus enzyme concentration ([*E*]). Each experiment consisted of three replicates.

### Biochemical characterization of FsAlgB

The effects of temperatures (20–80 °C) on the purified FsAlgB in 50 mM Tris-HCl buffer of pH 8.0 were investigated at pH 8.0. The thermal stability of the enzyme was determined under the standard assay conditions after incubating the purified FsAlgB at 20–80 °C for 30 min. In addition, the thermally induced denaturation was also investigated by measuring the residual activity after incubating the enzyme at 20–40 °C for 0–180 min. The effects of pH on the enzyme activity of FsAlgB were evaluated by incubating the purified enzyme in buffers with different pH (4.0–11.0) under the standard conditions. The buffers with different pHs used are 50 mM phosphate citrate (pH 4.0–5.0), 50 mM NaH_2_PO_4_-Na_2_HPO_4_ (pH 6.0–8.0), 50 mM Tris-HCl (pH 7.0–9.0), and glycine-NaOH (pH 9.0–10.0). The pH stability was characterized by determining the residual activity after the enzyme was incubated in buffers with different pH (4.0–11.0) for 24 h. Each experiment consisted of three replicates.

The influences of metal ions on the activity of FsAlgB were performed by incubating the purified enzyme in 50 mM Tris-HCl buffer of pH 8.0 at 4 °C for 24 h in the presence of various metal compounds with a final concentration of 1 mM. Then, the activity was measured under standard conditions and the reaction mixture without any metal ion was taken as 100%. The effects of NaCl on enzyme activity of FsAlgB were measured in buffers with different concentrations of NaCl (0–800 mM) and the maximal enzyme activity served as control. Each experiment consisted of three replicates.

### Action pattern and degradation product analysis

To determine the smallest substrate and identify the number of substrate-binding subsites of FsAlgB, hydrolysis reactions (10 μL reaction mixture) were performed at 40 °C for 24 h using FsAlgB in 50 mM Tris-HCl buffer of pH 8.0 and oligosaccharides (10 mg/mL) with different DPs (DP2–8). The degradation products were analyzed by ESI-MS in a positive-ion mode using the following settings: ion source voltage, 4.5 kV; capillary temperature, 275–300 °C; Tube lens, 250 V; sheath gas, 30 arbitrary units (AU); scanning mass range, 150–2000 m/z.

To determine the cleavage selectivity of the enzyme, the ESI-MS was used to analyze the degradation products in the hydrolysis procedure of FsAlgB toward sodium alginate, polyMG, polyM, and polyG, respectively. The reaction mixtures (800 μL containing 1 μg purified enzyme and 2 mg substrates) were incubated at 40 °C for 72 h. Then, the mixtures were centrifuged at 8000*g* rpm for 10 min to remove the undissolved materials. In addition, the hydrolysates were loaded onto a carbograph column (Alltech, Grace Davison Discovery Sciences, United Kingdom) to remove salts, and then, the eluate was concentrated, dried and re-dissolved in 1 mL methanol. The degradation products were then analyzed by TLC plate as previously reported [20]. In brief, the degradation products were developed by TLC plate (TLC silica gel 60 F254, Merck KGaA, Darmstadt, Germany) with the solvent system (1-butanol/acetic acid/water 3:2:3) and visualized by heating TLC plate at 130 °C for 5 min after spraying with 10% (v/v) sulfuric acid in ethanol. The ESI-MS was also employed to determine the composition of the hydrolysates using analysis conditions as described above.

### Molecular modeling and docking analysis

The three-dimensional structure of FsAlgB was constructed using Protein Homology/analogY Recognition Engine V 2.0 on the basis of homologues of known structure (alginate lyase A1-II′ from *Sphingomonas* sp. A1 with PDB ID: 2ZAB) with sequence similarity of 39%. The molecular docking of the FsAlgB and tetrasaccharide (GGGG) was performed using MOE (Molecular Operating Environment, Chemical Computing Group Inc., Montreal, Canada). The ligand-binding sites were defined using the bound ligand in the crystal structures. PyMOL (http://www.pymol.org) was used to visualize and analyze the modeled structure and to construct graphical presentations and illustrative figures.

## Results

### Sequence analysis of the alginate lyase gene

The gene FsAlgB was cloned and sequenced (accession number: MG063276). As shown in Additional file 1: Figure S1, the open reading frame (ORF) of FsAlgB consists of 900 bp and encodes a putative alginate lyase composed of 299 amino acids with a theoretical molecular mass of 34.50 kDa. According to the conserved domain analysis, FsAlgB possesses only a C-terminal catalytic domain consisting of 231 amino acids (Thr_54_–Glu_284_).

**Fig. 1:**
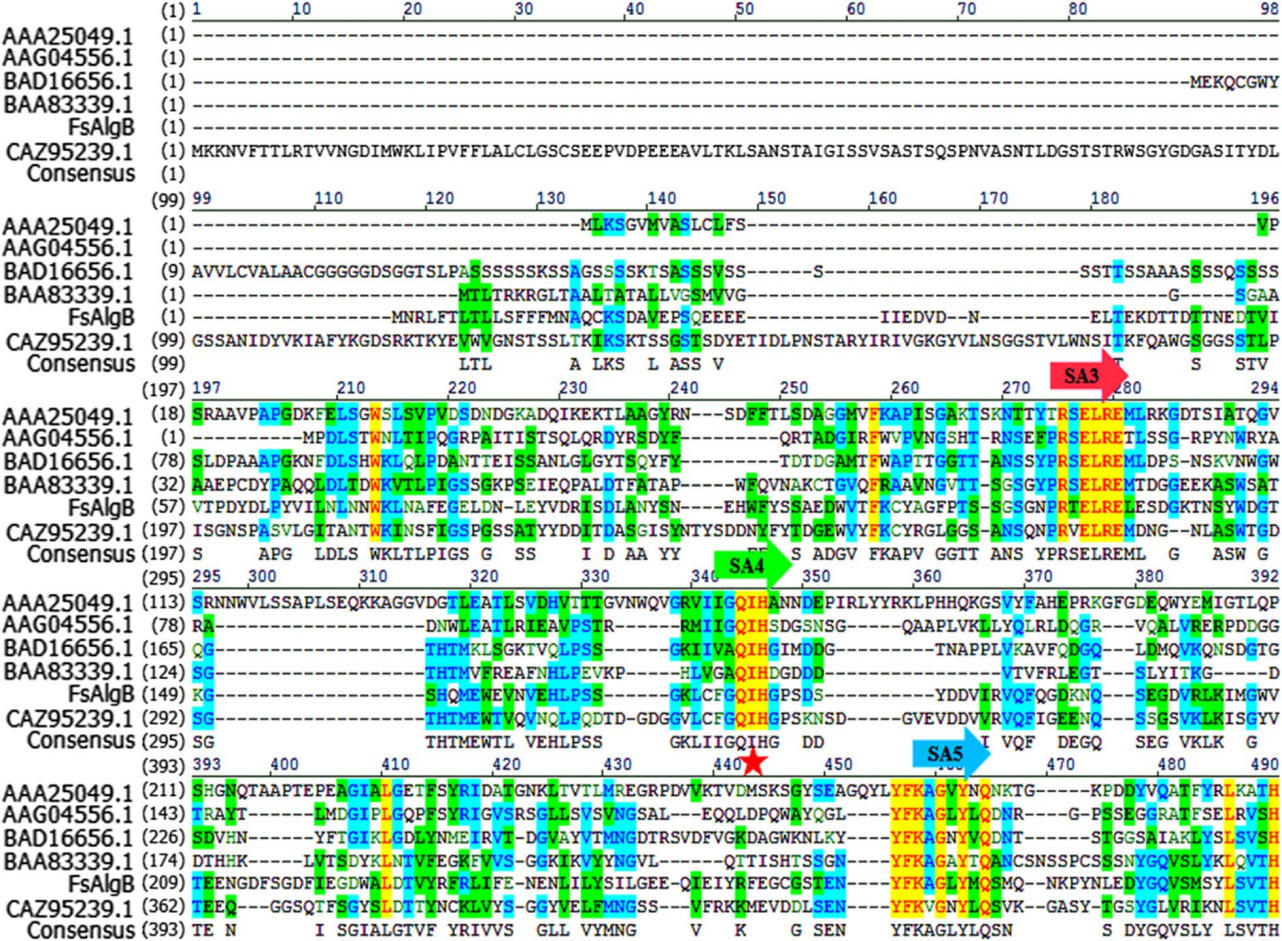
Multiple amino acid sequence alignment of FsAlgB and other alginate lyases of PL 7 family: AlyA (AAA25049) from *Klebsiella pneumoniae* subsp. *Aerogenes*, AlyA5 (CAZ96266) from *Zobellia galactanivorans* DsiJT, PA1167 (AAG04556) from *Pseudomonas aeruginosa* PAO1, AlyPG (BAA83339) from *Corynebacterium* sp. ALY-1, AlyA1 (CAZ95239) from *Zobellia galactanivorans* DsiJT, and A1-II′ (BAD16656) from *Sphingomonas* sp. A1. Identical and similar amino acid residues among the alginate lyases are shaded in yellow. The locations of three conserved regions are marked and the key amino acid “I” is marked with red star. SA3-5 indicated the common strands located in the sequence alignments

**Fig. 2:**
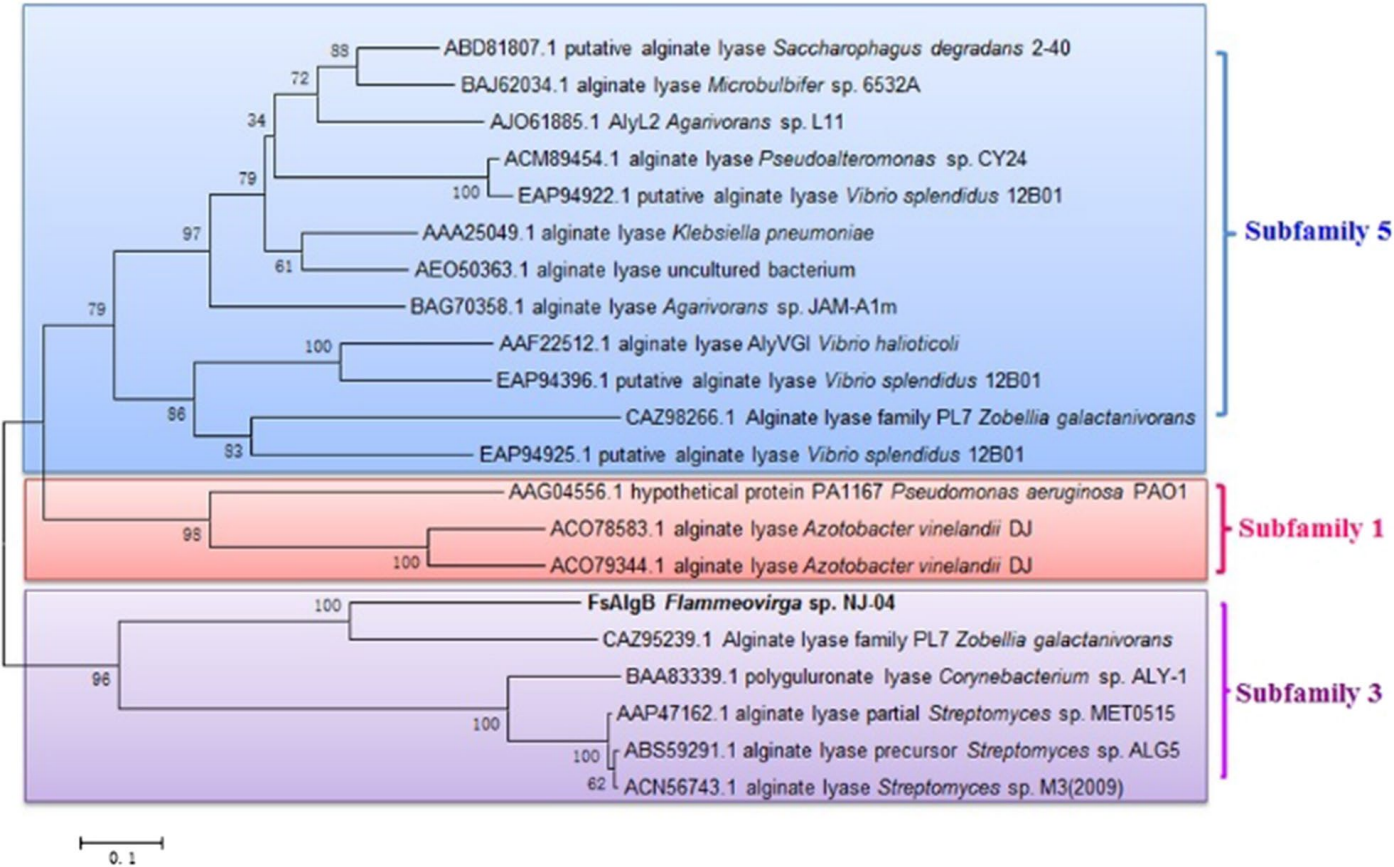
Phylogenetic tree of FsAlgB (the full-length enzyme) and other alginate lyases of PL 7 family based on amino acid sequence comparison. The species names are indicated along with accession numbers of corresponding alginate lyase sequences. Bootstrap values of 1000 trials are presented in the branching points

As shown in Fig. 1, FsAlgB shares the highest identity of 65% with FsAlgA from the same strain *Flammeovirga* sp. NJ-04 (GenBank accession no. ASA33934.1) [20] and exhibits rare similarity with the other characterized alginate lyases, which indicates that FsAlgB is a novel alginate lyase of PL 7. According to the analysis of amino acid sequence, FsAlgB contains the conserved regions such as “PRT/V/SELRE”, “YFKA/VGN/VY”, and “QIH” (as marked in Fig. 1), which contribute to the substrate binding and catalytic activity [18]. These conserved amino acid residues are all located in strand A3, A4, and A5 (as shown in Fig. 1), which is similar with the other alginate lyase of PL 7 family [18]. It has been reported that G block- and M block-degrading enzymes contain QIH and QVH in the conserved region, indicating that the amino acid residue I may recognize the polyG block or MG block [24]. To further determine the subfamily of FsAlgB, the phylogenetic tree (using the full-length sequence of FsAlgB) was constructed based on to compare the sequence homology (Fig. 2). It can be observed that FsAlgB clusters with representative enzymes of subfamily 3, and thus, FsAlgB is a member of the subfamily 3 alginate lyases.

### Cloning and expression of FsAlgB

**Fig. 3:**
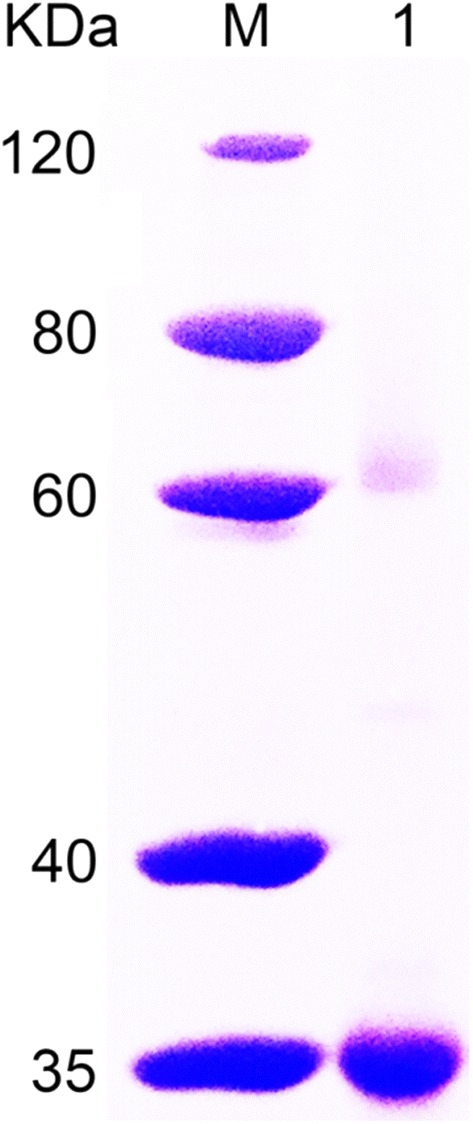
SDS-PAGE analysis of purified FsAlgB. Lane M protein: restrained marker (Thermo Scientific, USA); lane 1: purified FsAlgB

For further characterization, the alginate lyase FsAlgB was heterologously expressed in *E. coli* BL21 (DE3), followed by being purified by Ni–NTA Sepharose affinity chromatography and analyzed by SDS-PAGE. As shown in Fig. 3, a clear band (about 35 kDa) of purified FsAlgB can be observed at the gel, which is close to the predicted molecular mass of 34.50 kDa. Afterwards, four kinds of substrates (sodium alginate, polyMG, polyM, and polyG) were employed to investigate the substrate specificity of FsAlgB. As shown in Table 1, recombinant FsAlgB showed higher activity towards polyG (2445.6 U/mg) and polyMG (2103.2 U/mg) than to sodium alginate (1760.8 U/mg) and polyM (1124.9 U/mg). Thus, FsAlgB possesses broad substrate specificity.

**Table 1:** Substrate specificity and kinetics of FsAlgB (data shown are the mean ± SD, *n* = 3)

Substrate	Sodium alginate	polyMG	polyM	polyG
Activity (U/mg)	1760.8 ± 15.8	2103.2 ± 21.3	1124.9 ± 8.2	2445.6 ± 12.7
*K*_m_ (mM)	1.28 ± 0.28	0.92 ± 0.04	2.06 ± 0.17	0.69 ± 0.11
*V*_max_ (nmol/s)	0.12 ± 0.03	0.15 ± 0.01	0.08 ± 0.01	0.18 ± 0.04
*k*_cat_ (s^−1^)	2.22 ± 0.19	2.73 ± 0.23	1.48 ± 0.07	3.33 ± 0.21
*k*_cat_*/K*_m_ (s^−1^/mM)	1.73 ± 0.32	2.97 ± 0.41	0.72 ± 0.17	4.83 ± 0.39

The kinetics of FsAlgB towards sodium alginate, polyMG, polyM, and polyG were calculated according to the hyperbolic regression analysis. The non-linear fit curves for the degradation of the four kinds of substrates are shown in Additional file 1: Figure S2. As shown in Table 1, the *K*_m_ values of FsAlgB with sodium alginate, polyMG, polyM, and polyG as substrates were 1.28 mM, 0.92 mM, 2.06 mM, and 0.69 mM, respectively. Thus, FsAlgB had a much lower *K*_m_ values towards polyG and polyMG, indicating that it exhibited higher affinity towards G block than that to M block. The *k*_cat_ values of FsAlgB towards sodium alginate, polyMG, polyM, and polyG were 2.22 s^−1^, 2.73 s^−1^, 1.48 s^−1^, and 3.33 s^−1^, respectively. It indicated that FsAlgB exhibited the higher catalytic efficiency towards G block and hybrid MG block than that towards M block.

### Biochemical characterization of FsAlgB

**Fig. 4:**
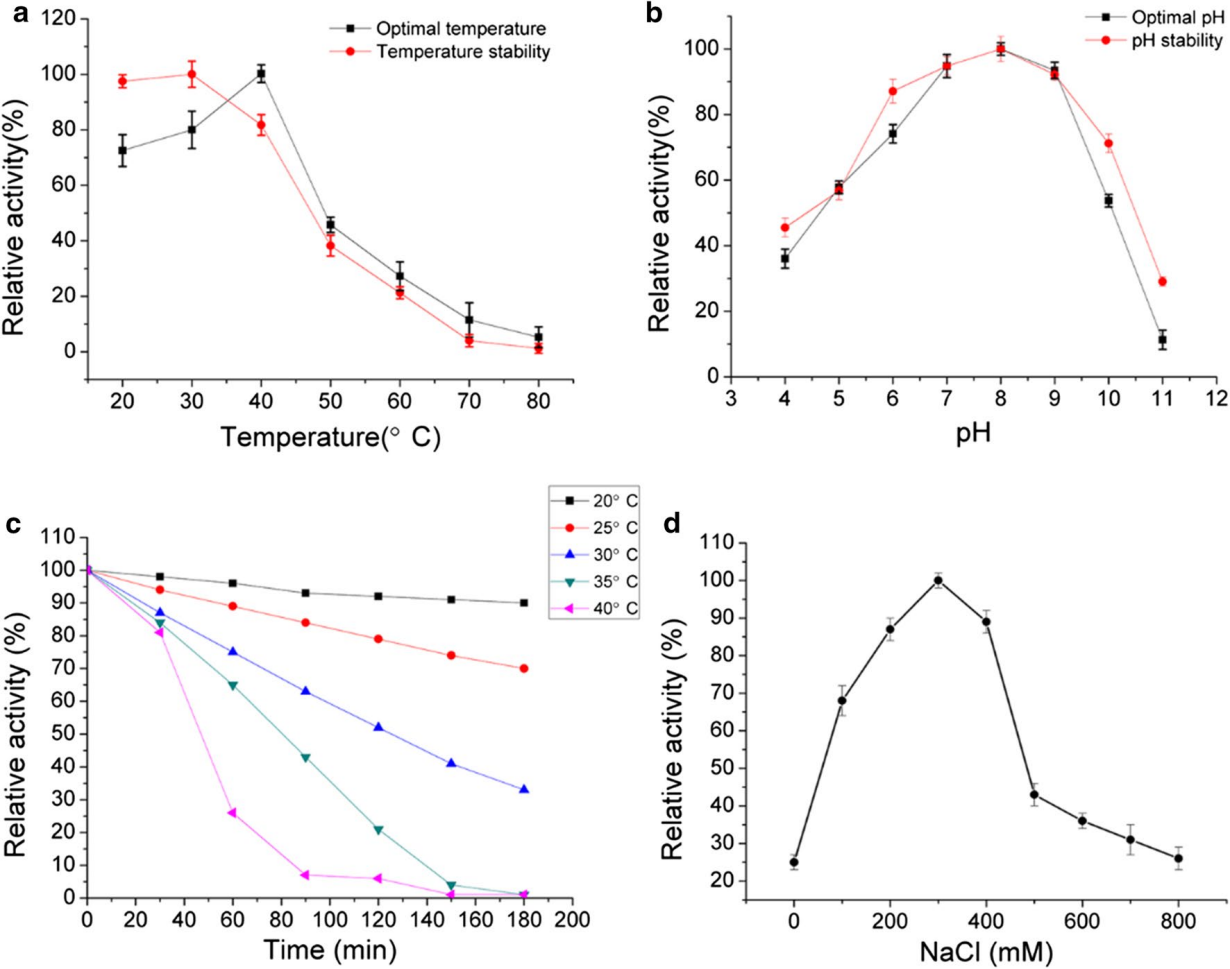
Biochemical characterization of FsAlgB. **a** The optimal temperature and thermal stability of FsAlgB. The thermal stability was evaluated by determining the residual activity after incubating enzyme at different temperatures for 30 min. The activity of the untreated enzyme was regarded as 100% and the relative activity was determined. **b** The optimal pH and the pH stability of FsAlgB. The pH stability was evaluated by determining the residual activity after incubating enzyme at different pH buffers for 24 h. The residual activity was measured in 50 mM Tris-HCl buffer (pH 8.0) at 40  °C. The maximal activity was regarded as 100% and the relative activity was determined. **c** The thermal-induced denaturation of FsAlgB. The residual activity was measured in 50 mM Tris-HCl buffer (pH 8.0) at 40  °C. **d** The effect of NaCl on enzymatic activity. The maximal activity in the test was relatively taken as 100%. The assay was measured in 50 mM Tris-HCl buffer (pH 8.0) at 40  °C (data shown are the mean ± SD, *n* = 3)

The biochemical characterization of FsAlgB was further performed. It showed maximal activity at 40 °C and was stable below 40 °C (Fig. 4a). The optimal pH of FsAlgB was 8.0 and the enzyme retained more than 80% activity after being incubated at a broad pH range of pH 6.0–9.0 for 24 h (Fig. 4b). However, this enzyme was mostly stable at pH 8.0. Thus, FsAlgB was an alkaline-stable lyase and it could retain stable in a broad pH range. This enzyme possessed approximately 80% activity after incubation at 40 °C for 30 min and was gradually inactivated as temperature increased (Fig. 4c).

The effects of metal ions on enzyme activity were also studied. As shown in Table 2 and Fig. 4d, the activity of FsAlgB can be activated by K^+^, Na^+^, and Ca^2+^ like other enzymes originated from the marine environment [24–28]. However, the activity can be inhibited by some divalent ions such as Zn^2+^, Cu^2+^, and Co^2+^.

**Table 2:** Effects of metal ions and chemical agents on FsAlgB activity (data shown are the mean ± SD, *n* = 3)

Metal ion	Relative activity (%)
Control	100 ± 2.1
K^+^	127.2 ± 2. 9
Mg^2+^	104.2 ± 3.2
Ca^2+^	109.2 ± 3.3
Cu^2+^	57.2 ± 2.0
Mn^2+^	97.3 ± 2.5
Ni^2+^	95.4 ± 3.4
Co^2+^	79.3 ± 4.8
Zn^2+^	46.7 ± 2.5

### Action pattern analysis and substrate docking of FsAlgB

**Fig. 5:**
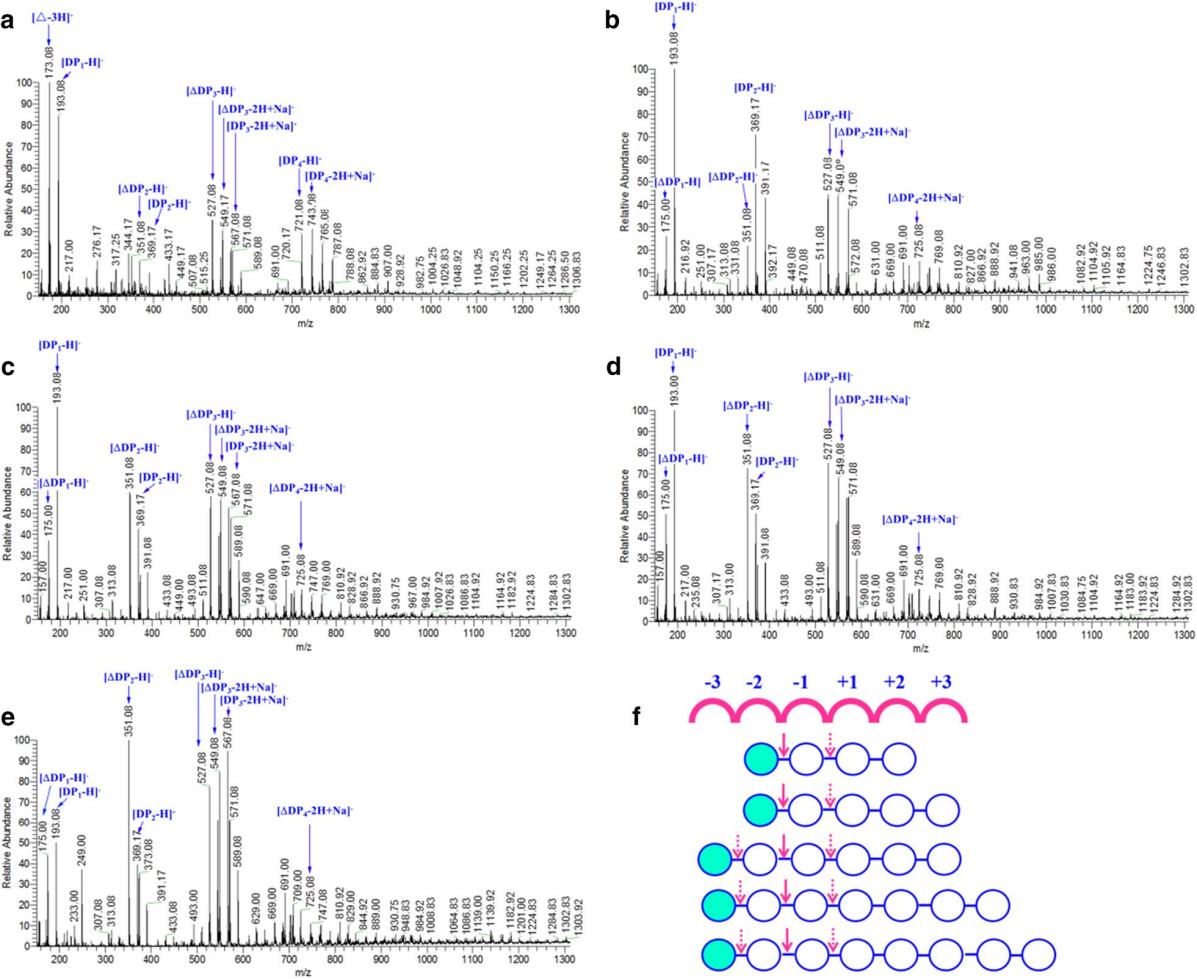
ESI-MS analysis of products with **a** tetrasaccharide, **b** pentasaccharide, **c** hexasaccharide, **d** heptasaccharide, and **e** octasaccharide as substrate. **f** Putative action pattern of FsAlgB towards different substrates. Arrows indicate possible cleavage sites of FsAlgB toward saturated oligosaccharide chains. Solid arrow represented prior cleavage, while dotted arrow represented secondary cleavage. ΔDP_*n*(*n* = 1–4)_ represents unsaturated oligosaccharides with DP of 1–4

To elucidate the action pattern and determine the number of substrate-binding subsites, we compared degradation capability of FsAlgB towards oligosaccharide with different DPs (DP2-8). As shown in Fig. 5, disaccharide and trisaccharide cannot be further degraded by FsAlgB with even higher concentration and longer incubation time (data not shown). Thus, tetrasaccharide was the shortest substrate that can be recognized and cleaved by FsAlgB, releasing monosaccharide, disaccharide, and trisaccharide (Fig. 5a). The degradation products of pentasaccharide, hexasaccharide, heptasaccharide, and octasaccharide were all similar, including oligosaccharides with DP of 1–4 (Fig. 5b–e). According to the results above, the putative bond cleavage of FsAlgB is depicted in Fig. 5f, it is presumed that FsAlgB can cleave the subsites between − 2 and + 1 within tetrasaccharide substrate to release mono-, di-, and trisaccharide, while, for penta-, hexa-, hepta-, and octasaccharide, they can be degraded into mono-, di-, tri-, and tetrasaccharide by cleaving the subsites of − 3 to + 1.

**Fig. 6:**
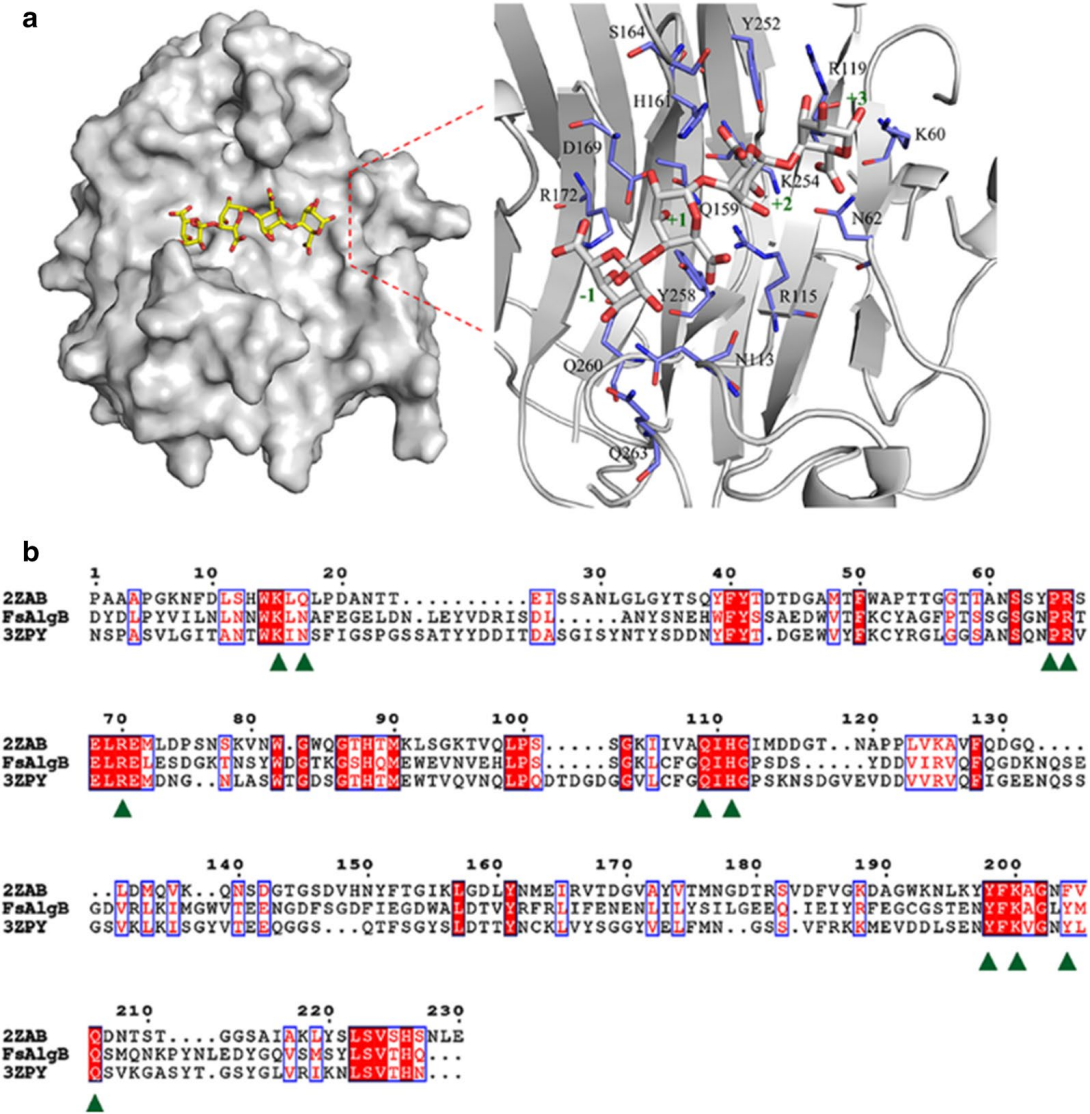
**a** Stereo view of the alginate tetrasaccharide (GGGG) bound to the tunnel-shaped active site of FsAlgB and **b** sequence alignments of FsAlgB and two alginate lyases with resolved structures (A1-II′ from *Sphingomonas* sp. A1 and AlyA1 from *Zobellia galactanivorans* DsiJT). The conserved key residues are marked with green triangles

The three-dimensional model of the FsAlgB was constructed based on the homologues structure of *Sphingomonas* sp. A1 alginate lyase A1-II′ (PDB ID: 2ZAB) with similarity of 39% using PHYRE2, and an alginate tetrasaccharide model (GGGG) was docked into the FsAlgB. Despite the low-sequence similarity between FsAlgB and A1-II′, the protein model was successfully constructed with 100% confidence, because the related proteins with divergent sequences share the same folding pattern of β-jelly roll [12]. As shown in Fig. 6a, the overall structure of the FsAlgB was predicted to fold into a β-sandwich jelly roll with two anti-parallel β sheets. The outer convex sheet includes five β-strands, and the inner concave sheet contains seven β-strands, forming a groove that harbors the catalytic active site. To identify the key residues for substrate recognition, the sequence alignment and protein–substrate interactions were analyzed. As indicated in Fig. 6b, the residues R_172_, Q_159_, His_161_, and R_119_ are highly conserved, and involved in the interaction between the protein and substrates in subsites − 1, + 1, + 2, and + 3, respectively. According to the docking, the residues Y_258_, Q_159_, H_161_, and R_119_ form hydrogen bonds with the carboxyl groups in subsites + 1, + 2, and + 3, respectively. According to the structural analysis, the active site of FsAlgB is located within two flexible loops (Fig. 6a). In addition, it has been reported that the flexibility of the two loops played an essential role in substrate recognizing and binding [18]. Both M and G in substrate molecules could be bound to the active site, and this structural characteristic provided basis for the broad substrate specificity of FsAlgB. Considering the docking results and the interaction between catalytic residues and tetrasaccharide substrate, it could be postulated that Q_159_ and R_119_ neutralized the negative charge of the carboxyl group, H_161_ abstracts the proton of C5, and Y_258_ donates proton to the glycoside bond to be cleaved.

### TLC and ESI-MS analysis of degradation products

**Fig. 7:**
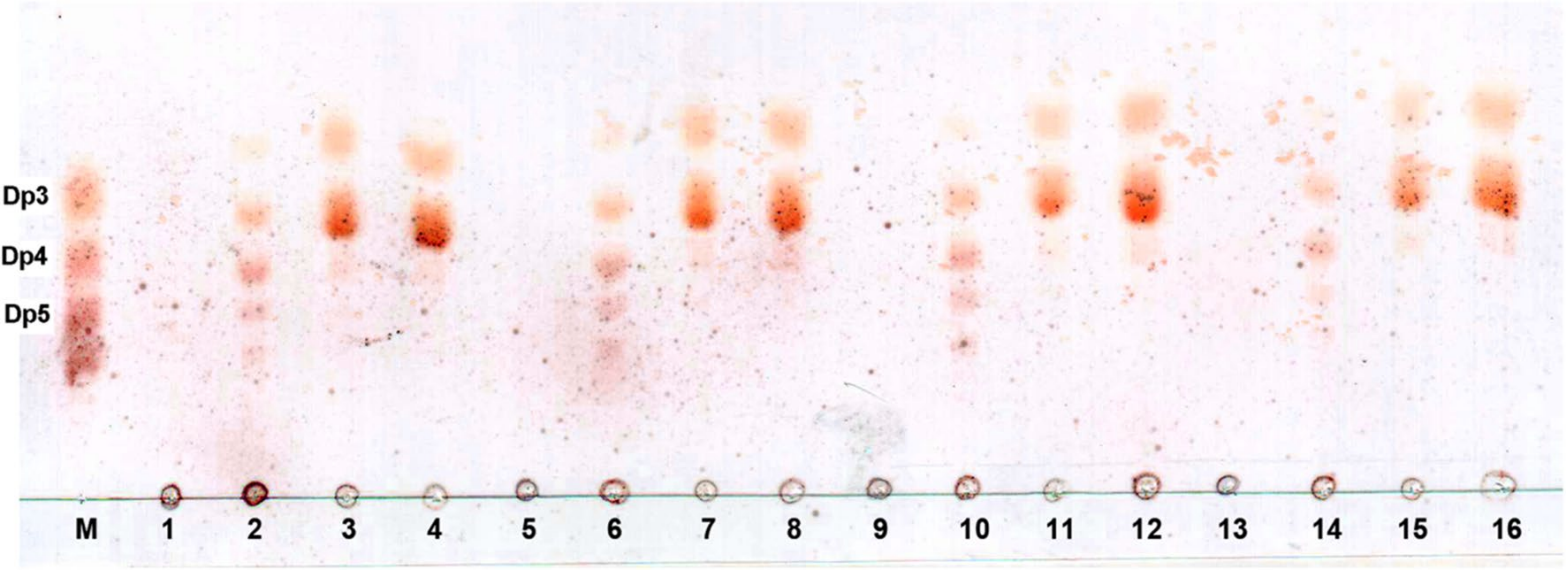
TLC analysis of the degradation products of FsAlgB. *Lanes 1*–*4* represent degradation products of alginate sodium for 0, 3, 12, and 24 h, *lanes 5*–*8* represent degradation products of polyMG for 0, 3, 12, and 24 h, *lanes 9*–*12* represent degradation products of polyM for 0, 3, 12, and 24 h, and *lanes 13*–*16* represent degradation products of polyG for 0, 3, 12, and 24 h. Lane M represents the oligosaccharide standards

**Fig. 8:**
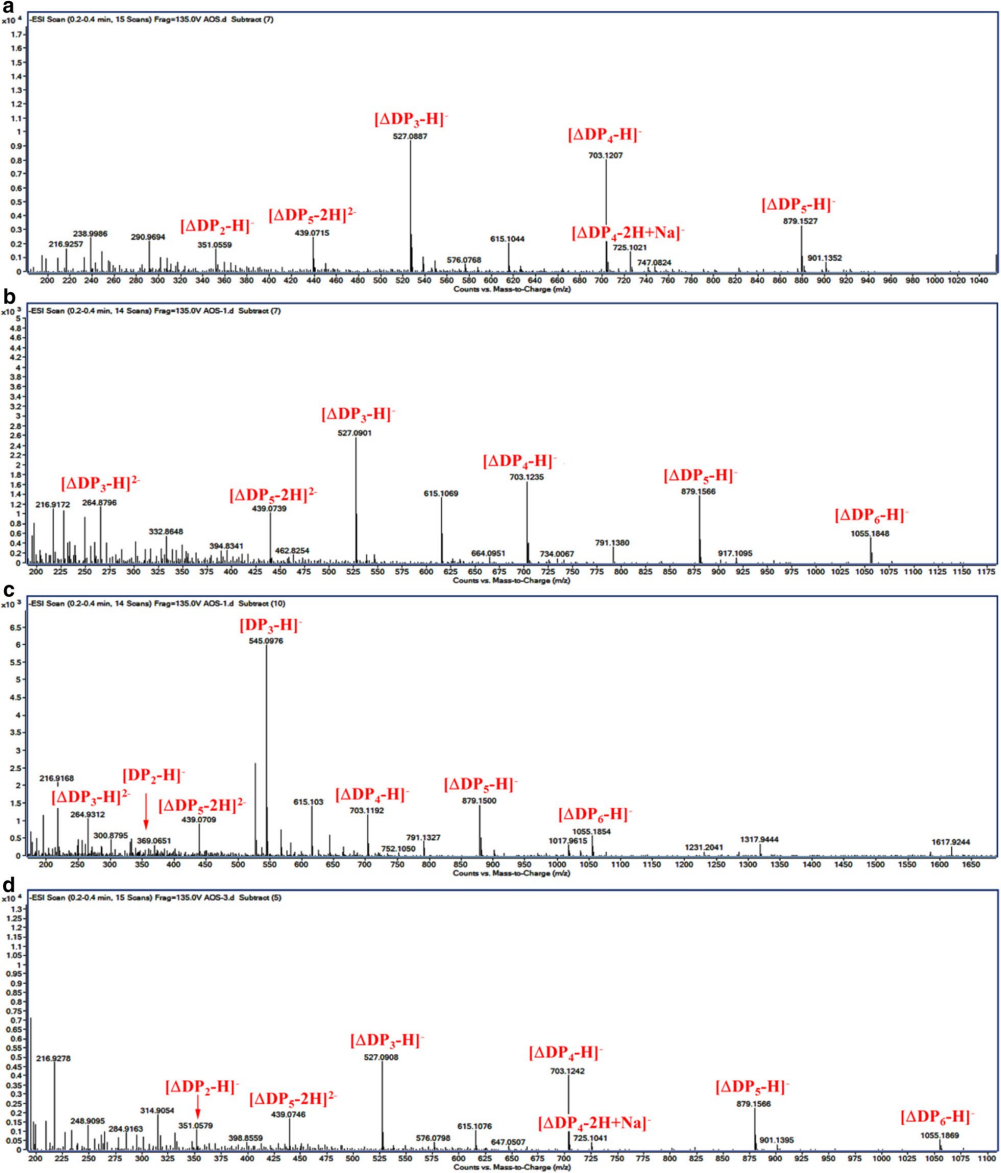
ESI-MS analysis of the hydrolysis products for 72 h with **a** alginate sodium, **b** polyMG; **c** polyM, and **d** polyG as substrate

The degradation products of FsAlgB towards four kinds of substrates for different times (0–24 h) were analyzed by TLC (Fig. 7). As the proceeding of degradation, oligosaccharides with various DPs (2–6) appeared. After incubation for 72 h, dimers and trimers turn to be main products. The results above indicate that FsAlgB can split the substrates in an endolytic manner. To further determine the composition of the degradation products, the hydrolysates were then analyzed by ESI-MS (Fig. 8). It can be observed that oligomers with DP of 2–5 were released with alginate and polyMG as substrate (Fig. 8a, b). While, for hydrolysates with polyM and polyG, the degradation products were trimers and tetramers with dimers as the main products, trimers, and tetramers accounting for a small fraction (Fig. 8c, d).

## Discussion

As described in our previous work, *Flammeovirga* sp. NJ-04 was previously isolated from South China Sea and exhibited versatile abilities for degrading algal polysaccharides. In addition, an endolytic alginate lyase, FsAlgA, with heat stability has been cloned and identified from the strain [20]. In this study, a new endo-type alginate lyase was identified from *Flammeovirga* sp. NJ-04 and its action pattern was further elucidated. The sequence alignment indicated that FsAlgB shares the highest identity of 65% with FsAlgA, which indicated that it is a new member of PL 7 family. In addition, it contained three conserved regions, namely “PRT/V/SELRE”, “YFKA/VGN/VY”, and “QIH”. Especially, the region of QIH was reported to be involved in substrate specificity; the residue “I” may recognize the polyG block or MG blocks [29]. Thus, it was reasonable that FsAlgB preferred polyG as the substrate just as other alginate lyases containing the QIH region, such as ALY-1 from *Corynebacterium* sp. strain ALY-1 [30], A1-I from *Sphingomonas* sp. A1 [31], and A1m from *Agarivorans* sp. JAM-A1m [32], while, for other alginate lyases containing the QVH region of PL 7 family, polyM is the optimal substrate, such as alginate lyaseA9m from *Vibrio* sp. JAM-A9m [24], alginate lyases AlyVOA, and AlyVOB from *Vibrio* sp. O2, degraded in activity assays [33].

While compared with the other PL 7 family enzymes, FsAlgB had lower *K*_m_ values. For instance, AlgMsp from *Microbulbifer* sp. 6532A possessed *K*_m_ values of 1.8–6.8 mM towards different substrates [22]. AlyA1 from *Zobellia galactanivorans* DsiJT showed *K*_m_ values varying from 1.7 mM to 6.2 mM [17]. The *k*_cat_/*K*_m_ values of FsAlgB towards polyG (4.83 mM^−1^ s^−1^) and polyMG (2.97 mM^−1^ s^−1^) were higher than to sodium alginate (1.73 mM^−1^ s^−1^) and polyM (0.72 mM^−1^ s^−1^), which indicated that the enzyme showed higher catalytic efficiency towards polyG and polyMG than to polyM.

As to biochemical characteristics, most of alginate lyases exhibit the optimal activity in the range of pH 7–8.5. For instance, FsAlgA from *Flammeovirga* sp. NJ-04 [20], AlyA1 from *Zobellia galactanivorans* [17], and Alg7D from *Saccharophagus degradans* [10] show the optimal activity at pH 7.0, while AlgMsp from *Microbulbifer* sp. 6532A [22], Algb from *Vibrio* sp. W13 [25], and A1-II′ from *Sphingomonas* sp. A1 [33] possess the optimal pH of 8.0. As to the optimal temperature, most of alginate lyases exhibit the optimal activity around 30–40 °C, while FlAlyA from *Flavobacterium* sp. UMI-01 had a highest optimal temperature of 55 °C [21]. In addition, AlgMsp from *Microbulbifer* sp. 6532A [22] and Alg7D from *Saccharophagus degradans* [10] displayed their maximal activity at 50 °C. Moreover, they could maintain most of the activities below 40 °C, except the Alg7D from *Saccharophagus degradans* which showed lower heat stability and retained only 40% of its maximum activity below 30 °C.

The effects of metal ions on FsAlgB were similar to that on Aly510-64 from *Vibrio* sp. 510-64 [26] and AlyAL-28 from *Vibrio harveyi* AL-28 [27]. Specifically, the activity of FsAlgB can be enhanced by NaCl with different concentrations (100–600 mM) and the maximal activity can be reached with 300 mM NaCl (Fig. 4d). Therefore, FsAlgB is a salt-activated alginate lyase. Since these salt-activated enzymes are usually isolated from marine bacteria, it is easy to understand the activation of NaCl [28]. Furthermore, it is assumed that high ionic strength might be essential to maintain the uronic acid units at a minimal interunit period for proper fitting of the enzyme [34]. The enhanced effects of NaCl with high concentration may be partly caused by the removal of bound water from alginate molecules or the stabilization of the transition state [27].

As to the action mode, ALY-1 from *Corynebacterium* sp. ALY-1 could degrade oligosaccharides larger than pentasaccharides, indicating that it possessed a subsite corresponding to hexasaccharide unites. The action pattern of the enzyme towards hexamers was confirmed by the HPLC analysis, and the main products were identified to be dimers and tetramers, which suggested that the catalytic site of the enzyme was matched to the linkage the second (− 2) and the third (− 1) residues from the nonreducing end [35]. Aly272 from *Alteromonas* sp. No272 exhibited no activity towards trimeric mannuronate or guluronate, but can degrade oligosaccharides larger than tetramers. The kinetic analysis *k*_cat_/*K*_m_ combined with intrinsic reaction rate constant (*k*_int_) and intrinsic substrate-binding constant (*k*_int_) indicated that it most likely consisted of six binding sites [36]. Almost all alginate lyases of PL7 family are endolytic enzymes and can degrade the alginate into oligosaccharides with low DP of 2–5 as the main product. Exceptionally, AlyA5 from *Zobellia galactanivorans* can release disaccharides in an exolytic manner [17]. Due to various biological activities, alginate oligosaccharides have been widely used in various fields [37, 38]. While considering the drawbacks and defects of chemical method, the alginate lyases, especially the enzymes with broad substrate specificity and high activity, are crucial in producing functional oligosaccharides. FsAlgB is a novel alginate lyase with broad substrate specificity and high activity, which can effectively degrade alginate and produce oligosaccharides with lower DPs, so it may be a potent tool to produce oligomers. Furthermore, this work also provided extended insights into the substrate recognition and degrading pattern of alginate lyases with broad substrate specificity.

## Conclusions

In this study, a novel bifunctional alginate lyase FsAlgB has been identified from *Flammeovirga* sp. NJ-04, which exhibited the highest activity (1760.8 U/mg) at pH 8.0 and 40 °C. In addition, it possessed broad substrate specificity, showing high activities towards not only polyM (polyβ-d-mannuronate) but also polyG (poly α-l-guluronate). And for further applications in industry, its action mode was also elucidated. Remarkably, FsAlgB can recognize the tetrasaccharide as the minimal substrate and cleave the glycosidic bonds between the subsites of − 3 and + 1 within the polysaccharide producing oligosaccharides with DP of 2–4. TLC and ESI-MS analysis indicated that it can degrade the substrates in an endolytic manner to release a series of oligosaccharides. Thus, with high activity, broad substrate specificity, and product specificity, it might be a potential candidate for degrading alginate to produce oligosaccharides.

## Additional file

**Additional file 1: Figure S1.** Nucleotide and deduced protein sequence of FsAlgB. The conserved catalytic domain (Thr_54_–Glu_284_) is marked with red box. **Figure S2.** Non-linear fit curves for the degradation of sodium alginate (A), polyMG (B), polyM (C), and polyG (D) by FsAlgB. The initial rates were determined with 0.1–10 mg/mL of each substrate at 40 °C. The data represent the mean of three experimental repeats with SD ≤ 5%.

## Abbreviations

DP: degree of depolymerization
PolyM: polyβ-d-mannuronate
PolyG: polyα-l-guluronate
SDS-PAGE: sodium dodecyl sulfate polyacrylamide gel electrophoresis
