# Supplementary material for: Elucidation of degrading pattern and substrate recognition of a novel bifunctional alginate lyase from Flammeovirga sp. NJ-04 and its use for preparation alginate oligosaccharides
Source: Biotechnol Biofuels. 2019 Jan 10;12:13. doi: 10.1186/s13068-019-1352-8 (PMC6327446; doi:10.1186/s13068-019-1352-8)
Supplement: Supplementary file 1 — Additional file 1: Figure S1. Nucleotide and deduced protein sequence of FsAlgB. The conserved catalytic domain (Thr54–Glu284) is marked with red box. Figure S2. Non-linear fit curves for the degradation of sodium alginate (A), polyMG (B), polyM (C), and polyG (D) by FsAlgB. The initial rates were determined with 0.1–10 mg/mL of each substrate at 40 °C. The data represent the mean of three experimental repeats with SD ≤ 5%. [file 13068_2019_1352_MOESM1_ESM.docx]

**Additional File**

**
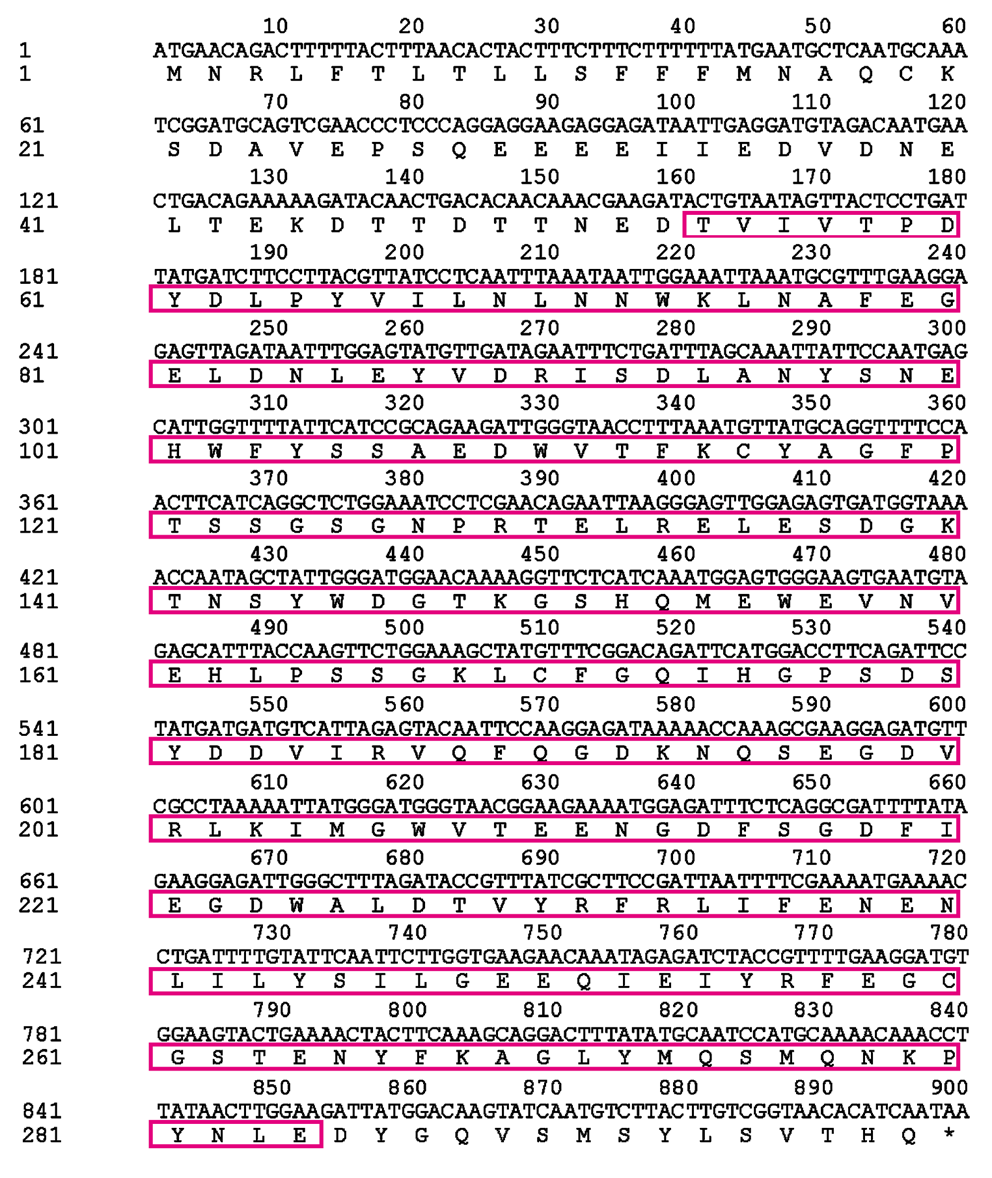
**

**Figure S1.**Nucleotide and deduced protein sequence of FsAlgB. The conserved catalytic domain (Thr_54_–Glu_284_) is marked with red box.


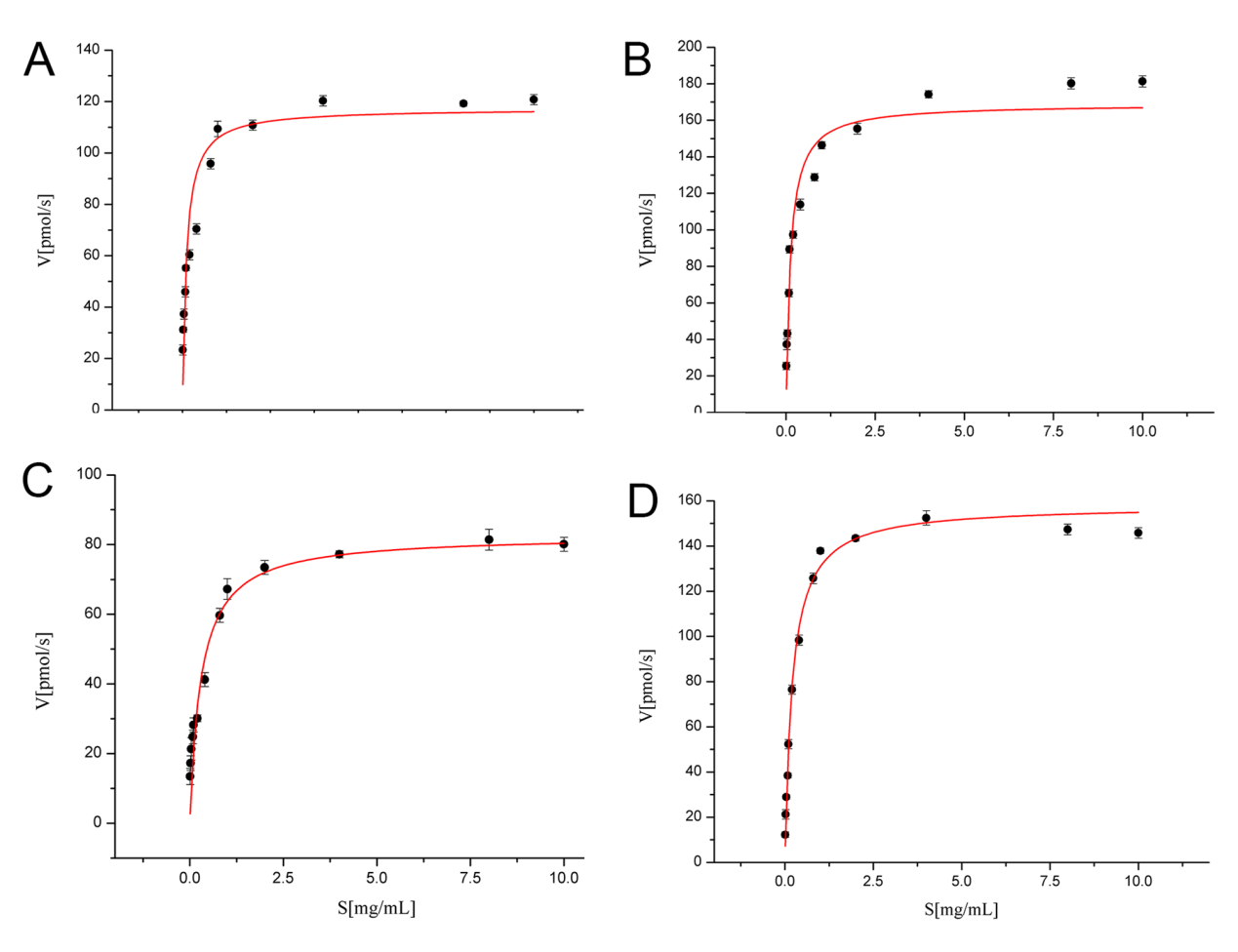


**Figure S2.** Non-linear fit curves for the degradation of sodium alginate (A), polyMG (B),

polyM (C) and polyG (D) by FsAlgB. The initial rates were determined with 0.1–10 mg/mL of each substrate at 40 °C. The data represent the mean of three experimental repeats with SD ≤ 5%.
